# Endoscopic Transmural Drainage of Pancreatic Pseudocysts: Technical Challenges in the Resource Poor Setting

**DOI:** 10.1155/2013/942832

**Published:** 2013-11-27

**Authors:** Shamir O. Cawich, Trevor Murphy, Sundeep Shah, Phillip Barrow, Milton Arthurs, Michael J. Ramdass, Peter B. Johnson

**Affiliations:** ^1^Department of Clinical Surgical Sciences, University of the West Indies, St. Augustine Campus, St. Augustine, Trinidad and Tobago; ^2^Department of Medicine, University of the West Indies, Mona Campus, Kingston, Jamaica; ^3^Departments of Surgery, Radiology, Anaesthesia, and Intensive Care, University of the West Indies, Mona Campus, Kingston, Jamaica

## Abstract

Although surgical drainage of pancreatic pseudocysts has been superseded by less invasive options, the requirement for specialized equipment, technical expertise, and consumables limits the options available in low resource settings. We describe the challenges experienced during endoscopic transmural drainage in a low resource setting and the methods used to overcome these barriers. Despite operating in a low resource environment, endoscopic drainage of pancreatic pseudocysts can be incorporated into our armamentarium with minimal change to the existing hardware. Careful patient selection by a dedicated multidisciplinary team should be observed in order to achieve good outcomes.

## 1. Introduction

Disruption of the pancreatic ducts can produce an acute fluid collection that matures to become surrounded by a fibrous capsule due to the chronic inflammatory reaction. The resulting pancreatic pseudocyst [1–3] may become complicated by haemorrhage, intestinal obstruction, infection, or rupture.

When complications develop, pseudocysts require some form of drainage [3, 4]. The traditional open surgical approach has now been superseded by less invasive options such as percutaneous drainage [3] or endoscopic drainage [4]. The advantages of these less invasive options are balanced by the need for technical expertise, specialized equipment, and increased cost. Therefore, these options are not universally available in low resource settings in developing countries [3, 5].

We report the challenges encountered in the low resource setting when performing endoscopic cystogastrostomy for pancreatic pseudocysts. To the best of our knowledge this is the first report of endoscopic cystogastrostomy from the Anglophone Caribbean.

## 2. Report of a Case

A 14-year-old boy presented to the emergency department eight weeks after being kicked in the epigastrium during a football match. He reported getting worse and nonbilious vomiting associated with an enlarging, tender epigastric mass. He could only tolerate small volumes of fluids orally.

On presentation he was mildly dehydrated, afebrile, and anicteric. There was upper abdominal distention associated with a firm epigastric mass. The mass was tender on deep palpation but there was no guarding or rebound tenderness. Bowel sounds were normal and a succussion splash was not present. The respiratory and cardiovascular examinations were normal.

**Figure 1:**
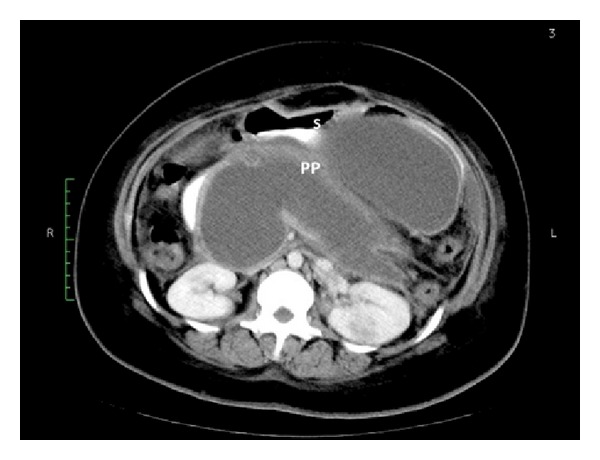
Axial slice of a CT scan of a patient with a large pancreatic pseudocyst (PP) demonstrating its apposition onto the posterior wall of the body of the stomach (S).

Liver function tests and serum amylase were normal. Abdominal ultrasound revealed a 13 × 19 × 21 cm cystic mass occupying the entire lesser sac, interposed between stomach and pancreas. Multiphase computer tomographic scans confirmed the presence of a well-organized pancreatic pseudocyst displacing the stomach anteriorly and contained in a thick-walled mature capsule (Figure 1). Endoscopic retrograde pancreatography revealed no evidence of proximal strictures and was unable to demonstrate the connection between the ductal system and the pseudocyst.

**Figure 2:**
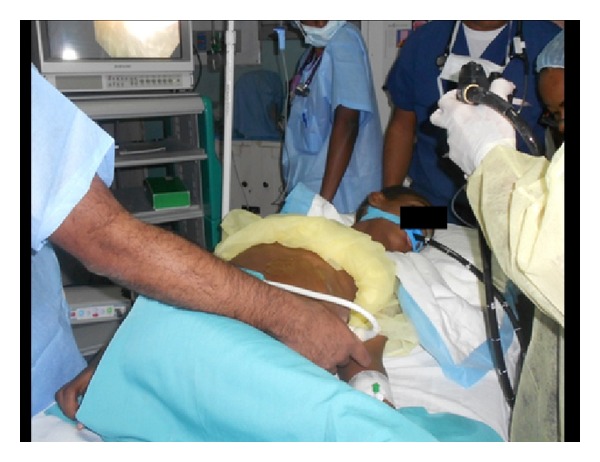
Gastroscope advanced into stomach to identify the area of bulging at the posterior gastric wall. Simultaneous transabdominal ultrasound being performed to guide the endoscopist to the ideal area for puncture.

**Figure 3:**
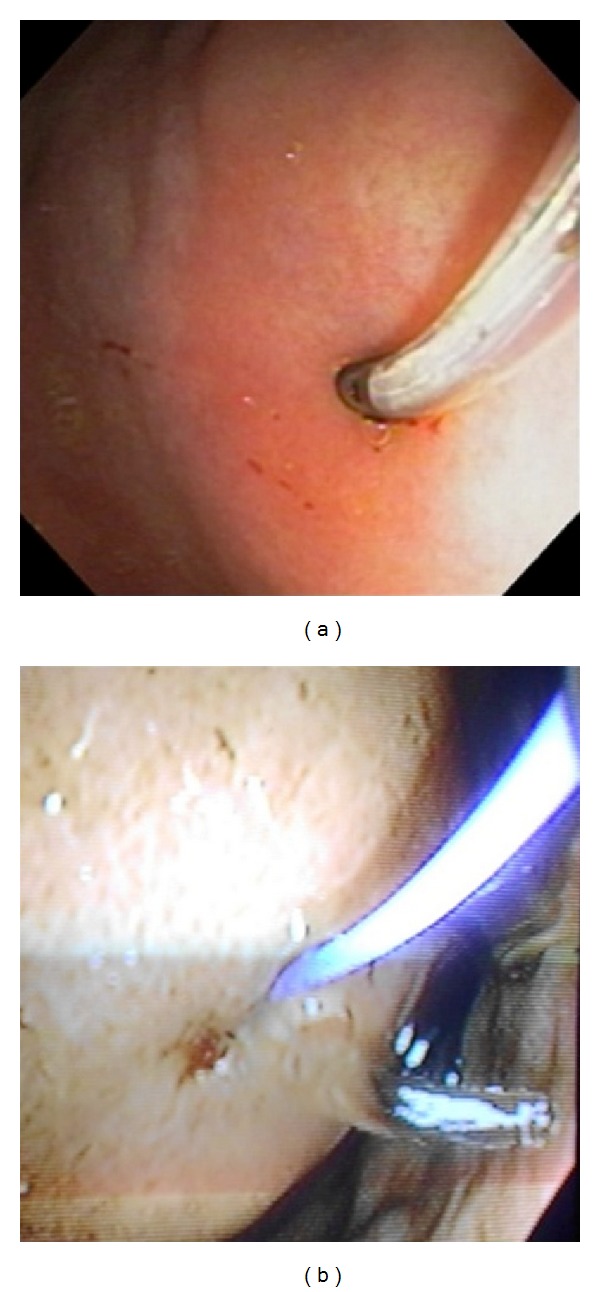
(a) A needle knife papillotome punctures the most protuberant point on the gastric mucosa; (b) entry into the cyst confirmed by a gush of clear fluid returning.

**Figure 4:**
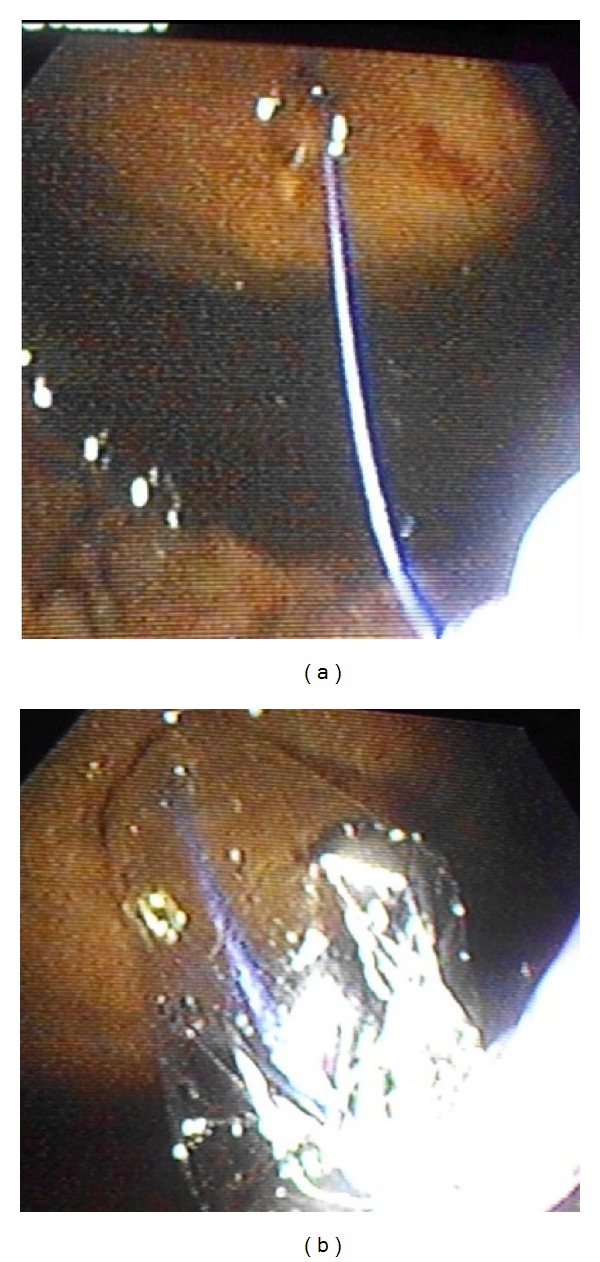
A balloon dilator was railroaded over a guidewire (a) to dilate the transmural tract to 16 mm (b).

**Figure 5:**
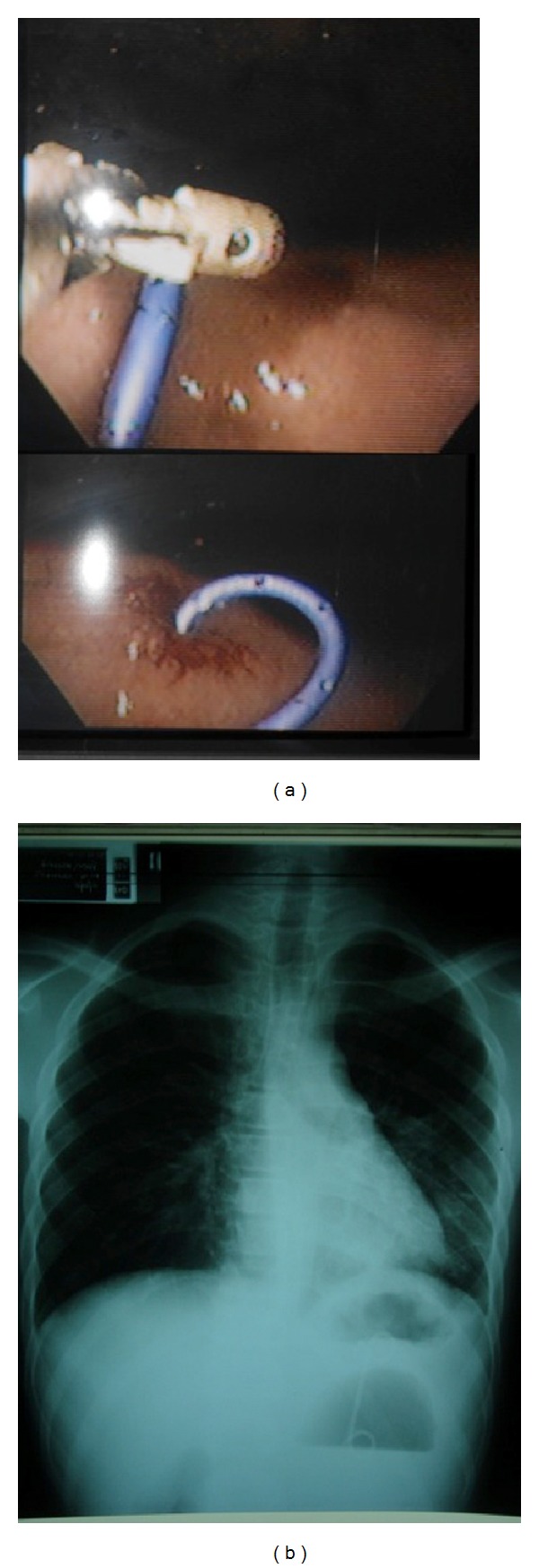
Placement of the end of the pigtail stent within the gastric lumen. Position confirmed on endoscopy (a) and on plan radiographs (b).

**Figure 6:**
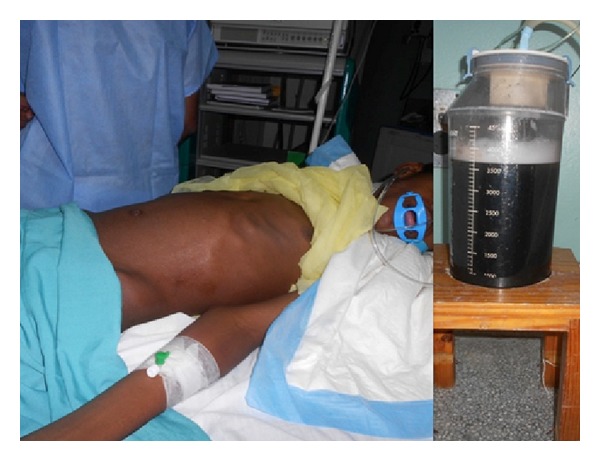
Immediate abdominal decompression (a) after 3700 mL of turbid pancreatic fluid was drained from the cyst (b).

An endoscopic cystogastrostomy was attempted in the endoscopy suite under conscious sedation with intravenous Propofol. The procedure was performed in the left lateral decubitus position with noninvasive monitoring. Intravenous ceftriaxone was administered as prophylaxis at induction. A side viewing duodenoscope (Olympus TJF-140, Olympus America, Central Valley, PA, USA) was advanced into the stomach. With insufflation the area of extrinsic gastric compression was identified on the posterior wall. Endoscopic ultrasound (EUS) was not available so the stomach was aspirated while the endoscope remained in situ. This facilitated simultaneous transabdominal ultrasound (Figure 2) to confirm that the endoscope tip was at an appropriate area for puncture. A triple lumen needle knife sphincterotome (Micro-knife XL, Boston Scientific Co., Marlborough, MA, USA) was advanced through the working channel of the scope and used to create a 1-2 cm incision in the gastric mucosa (Figure 3(a)) with bipolar electrocautery (force FX, Valleylab, Boulder, CO, USA). Entry into the cyst was confirmed by a gush of clear pancreatic fluid returning (Figure 3(b)). A 480 cm flexible 0.035′′ guidewire (Hydra Jagwire, Boston Scientific Co., Marlborough, USA) was advanced through the incision. The needle knife catheter was removed leaving the guidewire across the incision within the pseudocyst cavity. A 6–8 mm controlled radial expansion biliary dilating balloon (CRE Wireguided Balloon Dilator, Boston Scientific Microvasive, Natick, MA, USA) was railroaded over the guidewire (Figure 4(a)) and inflated to dilate the transmural tract to 16 mm (Figure 4(b)). The dilating balloon was inflated on three separate occasions for 20 seconds to ensure adequate dilation of the incision. The dilating balloon was removed with the guidewire left in place. A double pigtail 10 F × 5 cm plastic stent (C-flex Biliary; Boston Scientific, Spencer, IN, USA) was then advanced over the guidewire and deployed with the proximal end in the gastric lumen and the distal end within the pseudocyst cavity. Ultrasound was repeated to confirm drain placement within the cavity and the gastric placement was confirmed at endoscopy and subsequently with plain radiographs (Figure 5). Approximately 3700 mL of turbid pancreatic fluid was removed from the cyst, resulting in immediate abdominal decompression (Figure 6).

**Figure 7:**
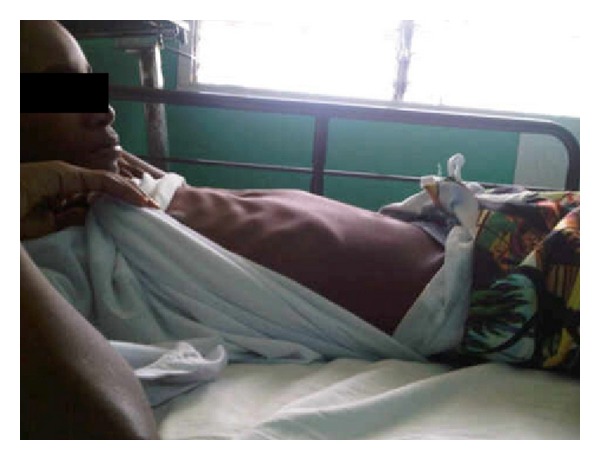
Abdomen remains flat one year after drainage.

The recovery period is uneventful and the patient was now able to tolerate a normal diet. The patient remained clinically well 6 months after stent removal (1 year after drainage) and the abdomen remained flat (Figure 7). Repeat ultrasound 12 weeks after stent removal showed no evidence of recurrence and the patient was discharged from followup.

## 3. Discussion

Open surgical drainage was first described in 1882 [6] but has become less popular over the past two decades as we witness a shift toward less invasive drainage procedures [4, 7, 8]. Laparoscopic drainage brings lower morbidity and faster recovery than open surgical drainage [8] but places an extra demand for specialized equipment and advanced laparoscopic skill sets that are not universally available in many developing countries. To date, there have been no reports of laparoscopic pseudocyst drainage from the Anglophone Caribbean.

Percutaneous drainage is feasible but it has low long-term success rates that range from 42% [9] to 50% [10], with percutaneous fistulae developing in 20% [7] to 40% [11] of cases. Therefore, percutaneous external drainage is relegated only to high-risk patients: patients with immature cyst walls unsuitable for internal drainage and those with infected cysts [3].

Endoscopic drainage was first described in 1985 [12] and quickly gained popularity because it avoided the need for general anaesthesia with relatively few complications. We performed a literature search in PubMed, MEDLINE, SCOPUS, SciELO, and Cochrane databases seeking reports on endoscopic drainage of pancreatic pseudocysts published in the past 5 years from June 2008 to June 2013. We excluded small studies with less than 15 drainage procedures. Twelve studies were identified reporting on EUS drainage in a total of 532 patients [13–24] with good clinical outcomes: technical success in 514 (96.6%) cases, clinical success in 500 (94.0%) cases, complications in 111 (20.9%) cases, and recurrence in 46 (8.7%) cases.

Two types of endoscopic drainage exist: transpapillary and transmural. Transpapillary drainage involves balloon dilation and stenting at endoscopic retrograde pancreatography, which should be done routinely to identify disruption or stenosis of the pancreatic ducts [7]. Successful transpapillary drainage requires a demonstrable communication between the pseudocyst cavity and main duct to allow for complete drainage [4, 7, 25]. Our patient was not a candidate for transpapillary drainage as there was no demonstrable communication present.

Transmural drainage can be done across the duodenal or gastric wall, depending on pseudocyst location. The prerequisites for transmural endoscopic drainage include <1 cm distance between the pseudocyst and intestinal wall on imaging, a clear impression of the intestinal wall at endoscopy, absence of varices, absence of pseudoaneurysms, and exclusion of malignant lesions before treatment [26–28].

Many endoscopists now perform transmural drainage under EUS guidance in order to avoid blood vessels [28–31] and select the optimal puncture site [32–37]. As EUS was not available in this low resource setting, we utilized transabdominal ultrasound to guide the puncture site. By removing the air interface from the insufflated stomach while leaving the scope in situ, we were able to visualize the scope at transabdominal ultrasound and determine its relationship to the pseudocyst cavity in order to avoid vessels and select the appropriate puncture site.

There is still lack of consensus on the need for routine EUS during transmural drainage procedures. During our literature search we encountered two prospective randomized trials comparing EUS and non-EUS guided drainage of pancreatic pseudocysts in a total of 90 patients [23, 24]. Overall, 45 patients who had EUS guided drainage were compared to 44 who had conventional drainage without EUS. Technical success was better in EUS guided studies (95.6%) than with conventional drainage without EUS (59.1%). Moreover, the 18 patients who had failed conventional drainage were crossed over into the EUS arm in both studies, with 100% success in these cases [23, 24]. Park et al. [24] reported that EUS guided and conventional drainage procedures had statistically similar complication rates (7% versus 10%). We eagerly await the results of the ongoing Cochrane review currently underway [38] designed to analyze the effectiveness of intramural endoscopic drainage with and without EUS.

Although there is evidence in support of EUS, there is still debate on whether it should be used routinely for transmural drainage. Yusuf and Baron [39] carried out a survey of 266 practicing gastroenterologists from the American Society of Gastrointestinal Endoscopy. They reported that 35% of endoscopists who regularly perform transmural drainage do not use EUS routinely, only employing this modality selectively [39]. There seems to be consensus, however, that EUS should be used in difficult cases where there is a small window for entry [12, 28], absent endoscopic bulge [39], unusual cyst location [12], or prior failed non-EUS guided attempts [12, 28].

Recent literature describes the use of a single step approach to transmural drainage where multiple drains are placed using specialized echoendoscopes with large diameter working channels [27, 28, 32–36]. However, in countries with limited resources, this type of specialized equipment is not usually available. We had no access to EUS or specialized echoendoscopes in this low resource setting. In fact, many of the consumables used were received as donations from charity organizations. The use of transabdominal ultrasound in this manner may be a low cost alternative to EUS and echoendoscopes for transmural endoscopic drainage in the resource poor setting. Although a drainage procedure can be successful when performed by an experienced dedicated multidisciplinary team, we acknowledge that careful patient selection should be exercised in the low resource setting.

## 4. Conclusion

Despite operating in a low resource environment, endoscopic drainage of pancreatic pseudocysts can be incorporated into our armamentarium with minimal change to the existing hardware. Careful patient selection and a dedicated multidisciplinary team are required to achieve good outcomes.
